# Polygalacturonase gene family analysis identifies *FcPG12* as a key player in fig (*Ficus carica* L.) fruit softening

**DOI:** 10.1186/s12870-023-04315-7

**Published:** 2023-06-14

**Authors:** Yuan Wang, Zhiyi Fan, Yanlei Zhai, Hantang Huang, Alexander Vainstein, Huiqin Ma

**Affiliations:** 1 College of Horticulture, China Agricultural University, Beijing, 100193 China; 2 The Robert H. Smith Faculty of Agriculture, Food and Environment, The Hebrew University of Jerusalem, Rehovot, Israel

**Keywords:** Fig (*Ficus carica* L.), Polygalacturonase gene family, Expression, Ethylene response factor, Fruit softening

## Abstract

**Background:**

The fig (*Ficus carica* L.) tree has high economic value. However, its fruit have a short shelf life due to rapid softening. Polygalacturonases (PGs) are essential hydrolases, responsible for the pectin degradation that plays a key role in fruit softening. However, fig *PG* genes and their regulators have not yet been characterized.

**Results:**

In this study, 43 *FcPG*s were identified in the fig genome. They were non-uniformly distributed on 13 chromosomes, and tandem repeat *PG* gene clusters were found on chromosomes 4 and 5. Ka/Ks calculation and collinear analysis indicated negative selection as the main driver of *FcPG* family expansion. Fourteen *FcPG*s were found expressed in fig fruit with FPKM values > 10, of which seven were positively correlated, and three, negatively correlated with fruit softening. Eleven *FcPG*s were upregulated and two downregulated in response to ethephon treatment. *FcPG12*, a member of the tandem repeat cluster on chromosome 4, was selected for further analyses due to its sharp increment in transcript abundance during fruit softening and its response to ethephon treatment. Transient overexpression of *FcPG12* led to decreased fig fruit firmness and increased PG enzyme activity in the tissue. Two ethylene response factor (ERF)-binding GCC-box sites were found on the *FcPG12* promoter. Yeast one-hybrid and dual luciferase assays showed that *FcERF5* binds directly to the *FcPG12* promoter and upregulates its expression. Transient overexpression of *FcERF5* upregulated *FcPG12* expression, thereby increasing PG activity and fruit softening.

**Conclusions:**

Our study identified *FcPG12* as a key *PG* gene in fig fruit softening, and its direct positive regulation by *FcERF5*. The results provide new information on the molecular regulation of fig fruit softening.

**Supplementary Information:**

The online version contains supplementary material available at 10.1186/s12870-023-04315-7.

## Introduction

Textural change is a characteristic and irreversible phenomenon of fruit ripening, accompanied by a series of other physiological and biochemical changes, such as sugar accumulation, color transformation, and aroma development. These changes result from the spatiotemporal expression of a series of fruit ripening-related genes [1]. The texture of the ripening fruit changes mainly due to pectin degradation, cell wall relaxation and expansion, and cellulose and hemicellulose disaggregation [2]. The decomposition of cell wall polysaccharides involves the synergistic effects of multiple enzymes and proteins, including polygalacturonase (PG), pectate lyase, endotransglycosylase/hydrolase, expansin, and others [3–5].

PG is one of the largest hydrolase families, belonging to glycoside hydrolase family 28 (GH28) [6, 7]. The *PG* gene family has been identified in many fruit species, including 45 *PG*s in sweet cherry (*Prunus avium*) [8], 51 *PG*s in kiwifruit (*Actinidia chinensis*) [9], 35 *PG*s in grape (*Vitis vinifera*) [10], 61 *PG*s in pear (*Pyrus pyrifolia*) [11], and 45 *PG*s in peach (*Prunus persica*) [12]; 66 *PG*s have been identified in *Arabidopsis* [6]. The *PG* family is widely involved in the cell wall modifications that are required for various tissues’ growth and expansion, such as shoots, leaves and roots, and for organ abscission. The transcription levels of different *PG*s are directly or indirectly regulated by internal and external factors, such as phytohormones, temperature and ultraviolet light [13].

*PG* family members are of interest in fruit development and ripening. Five *PG*s were identified as related to fruit development out of a family of 54 members in *Solanum lycopersicum* [14]. Two *PG*s were strongly related to fruit ripening in *Actinidia arguta*, and *AaPG18* was found to be involved in kiwifruit ripening processes [15]. In papaya (*Carica papaya*), *CpPG1* was revealed to play a key role in fruit softening [16]. The expression of *FaPG1* increased during strawberry fruit softening, and antisense *FaPG1* delayed softening [17]. In sweet cherry, transient overexpression of *PavPG38* reduced fruit firmness and the contents of ion-linked pectin (ISP) and covalent pectin (CSP) content, and increased the content of water-soluble pectin (WSP) [8].

The expression of *PG*s that are related to fruit ripening and softening is regulated by a number of transcription factors, including APETALA2/ethylene response factor (AP2/ERF) family transcription factors, the NAC family (NAM, ATAF1, ATAF2 and CUC2) and MADS-box family (MCM 1, AGAMOUS, DEFICIENS, SRF-box) [18, 19]. GCC-box, with core sequence AGCGCCC, is usually a *cis*-acting element that is directly bound by AP2/ERF protein on the target gene promoter [20]. In papaya, electrophoretic mobility shift assay demonstrated specific binding of *CpERF9* to the promoter of *CpPG5* via the GCC-box motif [21]. Knockout of *SlERF52* in tomato resulted in downregulation of *TAPG1*, *TAPG2* and *TAPG4* in the abscission layer [22]. In peach, *PpERF2* directly binds to the *PpPG1* promoter and inhibits *PpPG1* expression during fruit softening [23].

Fig (*Ficus carica* L.) is one of the earliest domesticated fruit trees. It is highly adaptable to planting environments, and produces considerable economic value [24]. The fig fruit is climacteric in nature, and fruit firmness rapidly decreases after ripening, making it extremely intolerant to storage and transportation, which hinders its use as a fresh fruit [25, 26]. The content and state of pectin and hemicellulose in the fig receptacle and flesh change when the fruit are physiologically ripe and begin to soften. Pectin solubilization is associated with higher activity of PG and pectin methylesterase (PME) enzymes [27]. The expression patterns of FcPG1 and FcPG2 have been isolated and identified, but in that study, the information on gene structure and sequence was incomplete, and the genes’ functions were not verified [28]. Thus, although the change in texture is an important biological characteristic that affects the shelf life of fresh figs, there is a lack of in-depth research on *PG* in figs, and the key *PG* in fruit ripening and softening has not yet been identified.

**Table 1:** Physical and chemical properties of FcPGs.

Gene locus	Gene name	Length (aa)	PI	MW (kDa)	Signal peptide
FCD_00006598	*FcPG1*	379	8.75	39608.37	+
FCD_00006599	*FcPG2*	392	9.82	42188.16	-
FCD_00006600	*FcPG3*	408	9.48	44112.18	-
FCD_00003245	*FcPG4*	439	9.44	47880.87	-
FCD_00035073	*FcPG5*	253	5.11	27577.1	+
FCD_00019240	*FcPG6*	404	8.32	42275.37	+
FCD_00027533	*FcPG7*	430	9.69	47535.61	-
FCD_00000864	*FcPG8*	468	6.52	50712.41	-
FCD_00027282	*FcPG9*	403	8.65	42166.08	+
FCD_00027283	*FcPG10*	395	9	41403.2	+
FCD_00027284	*FcPG11*	407	8.46	42422.43	+
FCD_00027285	*FcPG12*	400	8.73	41793.52	+
FCD_00027288	*FcPG13*	401	7.49	41922.56	+
FCD_00004402	*FcPG14*	474	8.9	51962.52	-
FCD_00001676	*FcPG15*	676	8.97	71911.06	-
FCD_00001748	*FcPG16*	441	5.99	47133.89	-
FCD_00014244	*FcPG17*	336	9.38	36776.96	-
FCD_00014247	*FcPG18*	401	4.86	43026.68	+
FCD_00014248	*FcPG19*	395	9.12	42465.46	+
FCD_00014249	*FcPG20*	394	8.76	41856.62	+
FCD_00014250	*FcPG21*	324	6.35	35119.76	+
FCD_00014251	*FcPG22*	397	8.71	41849.67	+
FCD_00014252	*FcPG23*	397	8.8	42045.02	+
FCD_00017446	*FcPG24*	280	5.97	30726.93	-
FCD_00007700	*FcPG25*	252	9.53	27539.78	-
FCD_00013341	*FcPG26*	421	9.38	45483.23	-
FCD_00013354	*FcPG27*	437	5.07	47049.82	+
FCD_00034636	*FcPG28*	352	7.54	38578.61	+
FCD_00010601	*FcPG29*	351	8.62	38620.16	-
FCD_00000097	*FcPG30*	404	8.61	43562.17	-
FCD_00000462	*FcPG31*	974	6.36	103843.5	+
FCD_00009441	*FcPG32*	437	8.76	47034.48	+
FCD_00023255	*FcPG33*	452	6.15	49040.19	-
FCD_00002804	*FcPG34*	423	8.86	46331.04	-
FCD_00002831	*FcPG35*	463	4.92	50464.81	+
FCD_00002832	*FcPG36*	277	6.57	30170.23	-
FCD_00007944	*FcPG37*	488	8.77	54518.7	+
FCD_00004971	*FcPG38*	483	5.3	52842.03	+
FCD_00021068	*FcPG39*	494	5.64	54262.8	+
FCD_00030337	*FcPG40*	483	7.14	52929.53	-
FCD_00032532	*FcPG41*	398	7.46	43500.42	+
FCD_00032533	*FcPG42*	501	8.35	55595.23	+
FCD_00037184	*FcPG43*	513	8.96	52545.14	+

**Fig. 1:**
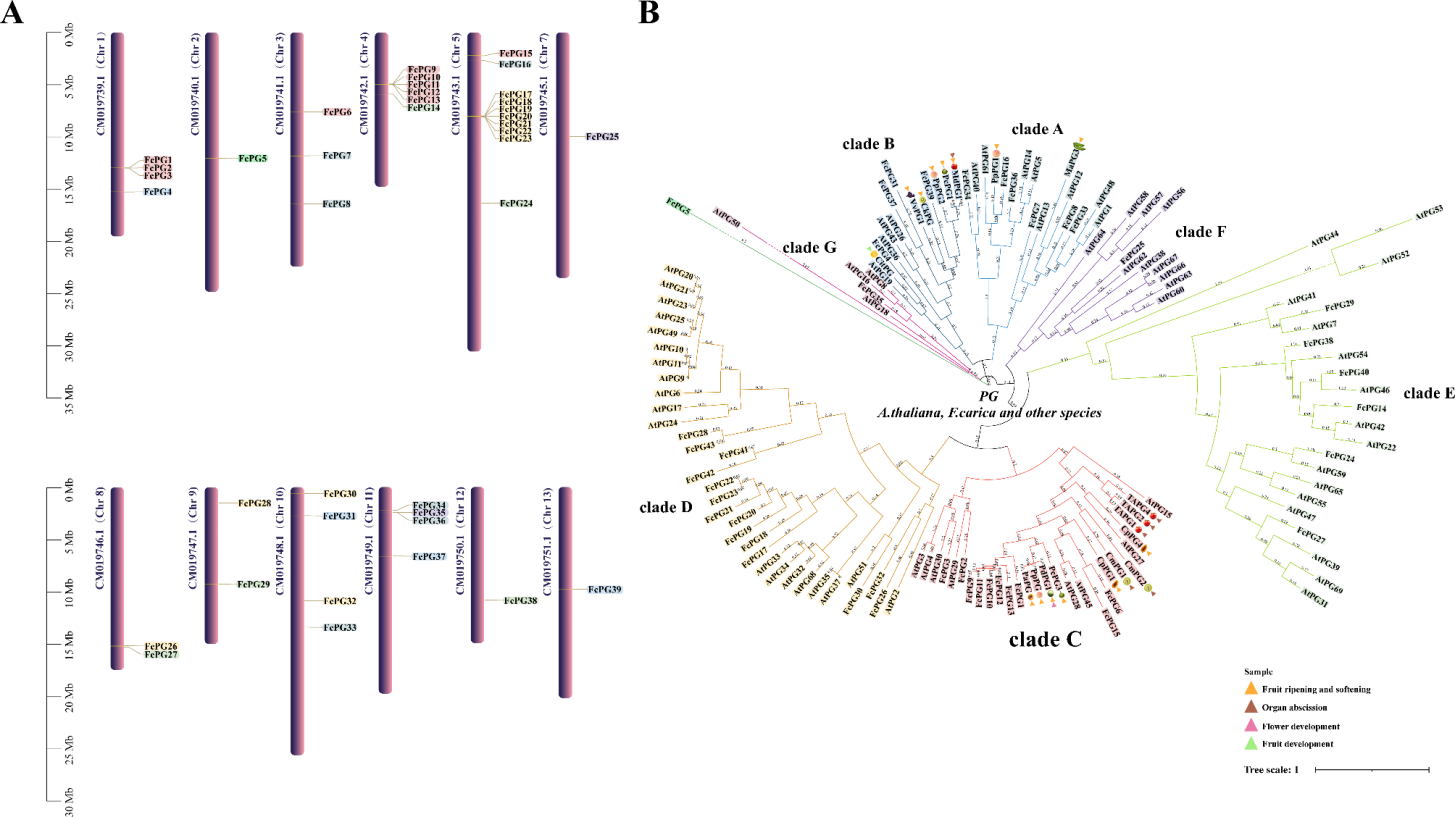
Chromosomal location and phylogenetic tree of *FcPG*s. (**A**) *FcPG*s are marked on chromosomes; scale bar on the left indicates length of fig chromosome (Mb). The color of the gene background corresponds to the clade color in the phylogenetic tree of panel B. (**B**) Phylogenetic tree showing the relationships of the 43 *FcPG*s in *F. carica*, and *PG*s in *A. thaliana* and other species. Four color triangles at the bottom right indicate identified biological functions of the *PG* genes

In this study, the *PG* gene family and *PG* expression in fig fruit were analyzed. *FcPG12* (FCD_00027285), a member of a small *PG* cluster on chromosome 4, was identified as a key *PG* in fruit softening. *FcERF5* was found to regulate *FcPG12*, activating its transcriptional expression and promoting fig fruit softening.

## Results

### Identification of PG gene family in *F. carica*

A total of 67 PG candidate sequences were obtained by pooling 65 sequences recruited using the *Arabidopsis* PG protein sequence as the query object; 56 sequences were screened by searching the GH28 HHM map of the fig genome database, and repetitions were deleted. After eliminating the protein sequences lacking *PG* domain characteristics by CDD database search and motif test, 43 PGs were identified. The *PG* genes were named *FcPG1 to FcPG43* according to their position on the chromosomes (Fig. 1A). The FcPGs were 252–974 amino acids long, with a calculated relative molecular mass of 2.75–10.38 kDa and a predicted pI of 4.86–9.82. About half of the FcPG proteins contained the signal peptide sequence predicted by SignalP (Table 1).

**Table 2:** Ka/Ks analysis of *FcPG* tandem repeat genes

Duplicated pair	Ka	Ks	Ka/Ks	Divergence time (MYA)
*FcPG1/FcPG9*	0.13	0.44	0.29	31.37
*FcPG1/FcPG10*	0.11	0.42	0.25	30.24
*FcPG1/FcPG11*	0.12	0.45	0.26	32.48
*FcPG1/FcPG12*	0.11	0.46	0.25	33.1
*FcPG1/FcPG13*	0.15	0.46	0.32	32.76
*FcPG12/FcPG9*	0.03	0.16	0.18	11.22
*FcPG12/FcPG10*	0.03	0.13	0.21	9.29
*FcPG12/FcPG11*	0.03	0.17	0.17	12.18
*FcPG12/FcPG13*	0.06	0.25	0.25	18.16
*FcPG9/FcPG10*	0.04	0.13	0.31	9.6
*FcPG9/FcPG11*	0.02	0.04	0.54	2.72
*FcPG9/FcPG13*	0.07	0.26	0.28	18.23
*FcPG10/FcPG11*	0.03	0.15	0.22	10.83
*FcPG10/FcPG13*	0.07	0.27	0.26	19.51
*FcPG11/FcPG13*	0.07	0.27	0.26	19.29

Ka/Ks < 1, negative selection; Ka/Ks = 1, neutral selection; Ka/Ks > 1, positive selection; MYA, million years ago

To clarify the evolutionary relationship and functional differences between *FcPG*s, a phylogenetic tree of fig, *Arabidopsis* and 19 *PG* genes identified in other plants was constructed by the ML method. The *FcPG*s could be divided into seven clades, of which clade D, with 14 genes, was the largest (Fig. 1B). *FcPG*s were non-uniformly distributed on the chromosomes. Two tandem repeat regions, including *FcPG9*, *10*, *11*, *12*, *13* and *FcPG17*, *18*, *19*, *20*, *21*, *22*, *23*, respectively, on chromosomes 4 and 5 (Fig. 1A) were separately located on clades C and D in the phylogenetic tree (Fig. 1B). The sequences of *FcPG1*, *9*, *10*, *11*, *12* and *13* were similar and among them, *FcPG9*, *10*, *11*, *12*, *13* clustered together on the chromosome. Moreover, they were closely clustered with *PG* genes functioning in fruit softening in apricot, plum, peach and pear. On clade B, *FcPG4*, *37*, *31* and *39* were also closely clustered with fruit softening-related *PG*s in grape, kiwifruit, peach, pear, apple and orange. Clade A, which clustered *FcPG34*, *16*, *36*, *7*, *8* and *33*, contained known *PG* genes of peach and banana (Fig. 1B).

The Ka/Ks ratios of *FcPG1*, *9*, *10*, *11*, *12* and *13* were 0.17–0.54; the values < 1 indicate that these genes were under purified selection, and the function of the encoded proteins was retained. The Ks of *FcPG1* was higher than that of the others (Table 2), suggesting that *FcPG1* was the first gene to be segregated and to differentiate. We estimate that the tandem duplication of *FcPG9*, *10*, *11*, *12* and *13* occurred 2.8–19.3 million years ago, whereas that of *FcPG1* occurred at least 10 million years earlier (Table 2).

### Gene structure, conserved motif and domain analysis of FcPGs

**Fig. 2:**
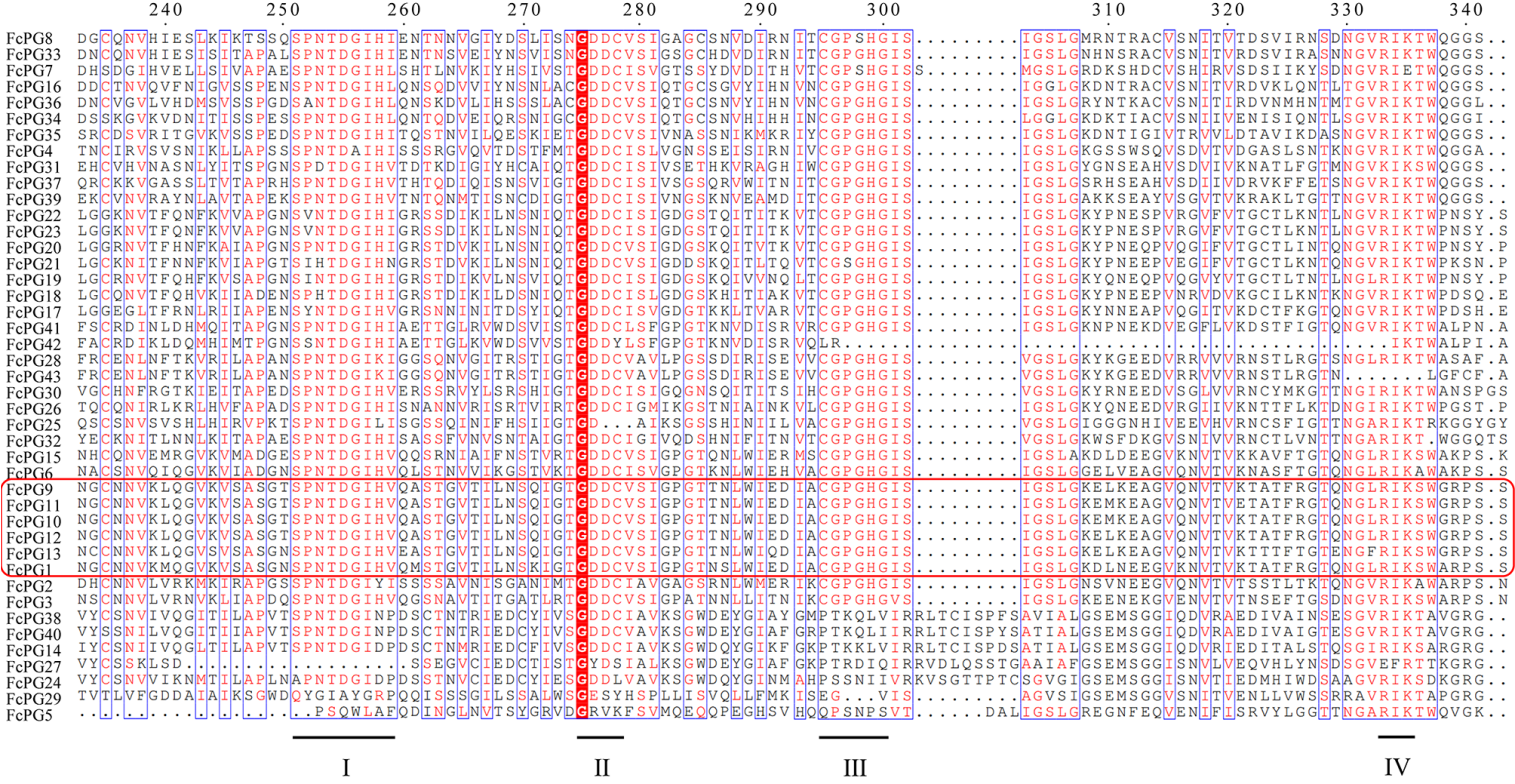
Multiple sequence alignments of the conserved domains (I–IV) of the FcPGs. Similar domains are indicated in red font and framed by blue boxes. Identical amino acid positions are highlighted by a red background. The gene cluster of *FcPG12* is circled in red

The amino acid sequence of PG proteins usually contains four conserved domains—SPNTDG, GDDC, CGPGHG and RIK, and three cysteine (Cys) sites. Multiple sequence alignment showed that most *FcPG*s have the four conserved domains (motifs I to IV) that are critical for PG’s hydrolytic activity. FcPG38, 40, 14, 27, 24, 29 and 5 lacked one or two of the typical PG domains (Fig. 2).

**Fig. 3:**
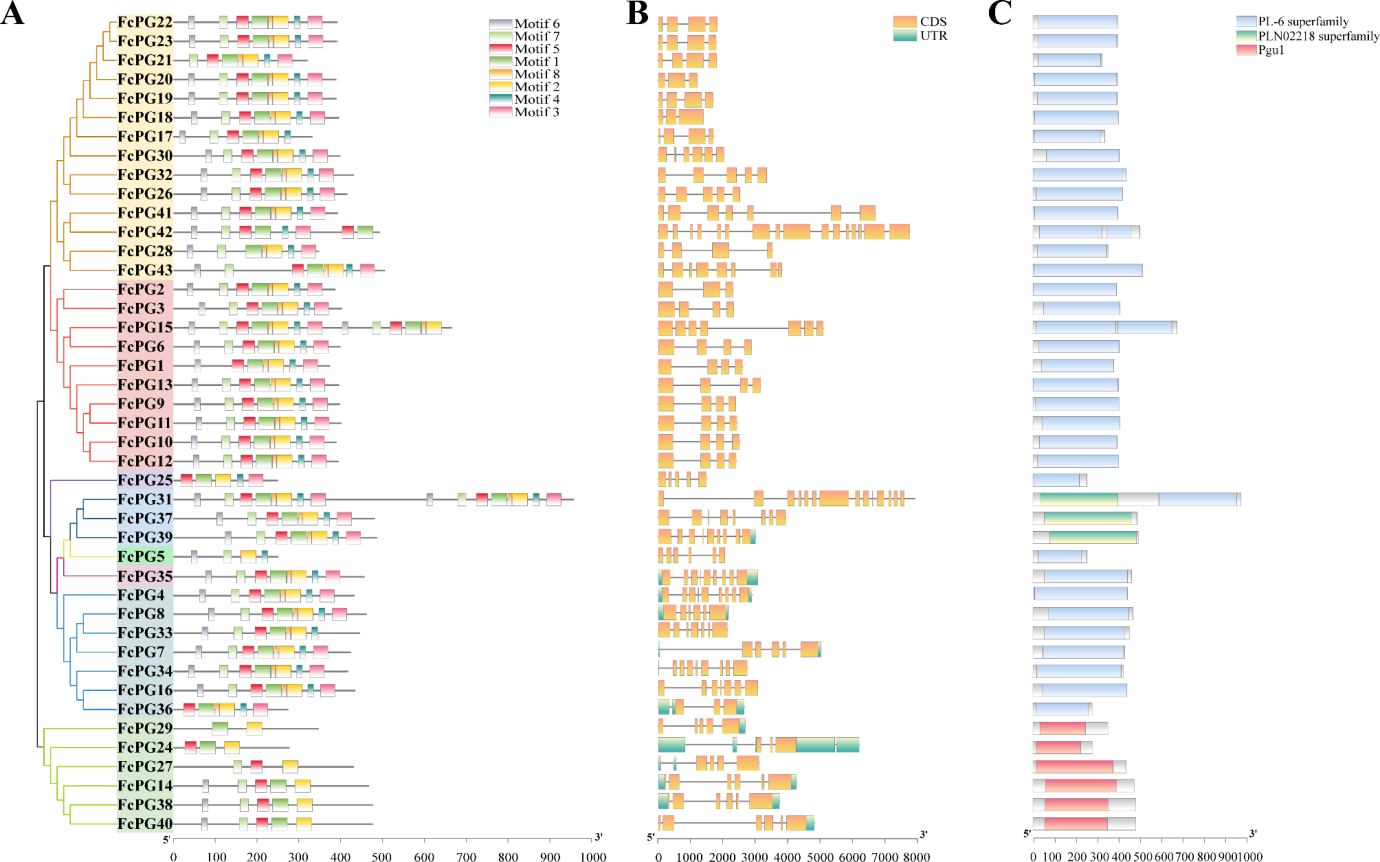
Structure of the *FcPG*s. (**A**) Motifs. (**B**) Gene structure. (**C**) Domains. The x-axis represents gene length. Gene background color corresponds to its clade color in Fig. 1B

Similar gene structure and composition were found for *FcPG* members of the same clade. *FcPG*s had 3–15 exons and 2–14 introns; fewer exons/introns were found in the members of clades A and G, whereas 4 exons/introns were identified in most other *FcPG*s (Fig. 3B). In addition, most of the *FcPG*s did not have a non-coding region structure; *FcPG*s with a non-coding region structure were mainly concentrated on clades E and F (Fig. 3B).

Eight conserved motifs were obtained when the 43 FcPG protein sequences were aligned (Fig. 3A). Within each clade of the phylogenetic tree, the composition and positional order of the conserved motifs were usually similar. Motifs 1 and 2 were present in all 43 PG protein sequences; motif 5 was absent in FcPG5 and FcPG29, and motif 3 was absent in members of clade F (Fig. 3A). According to the CDD database in NCBI, most of the PG protein domains belong to PL-6 (polysaccharide lyase family 6), the members on clade D belong to the PLN02218 superfamily, and the members on clade F are in the Pgu1 family (Fig. 3C).

### Expression patterns of FcPGs in fig fruit

**Fig. 4:**
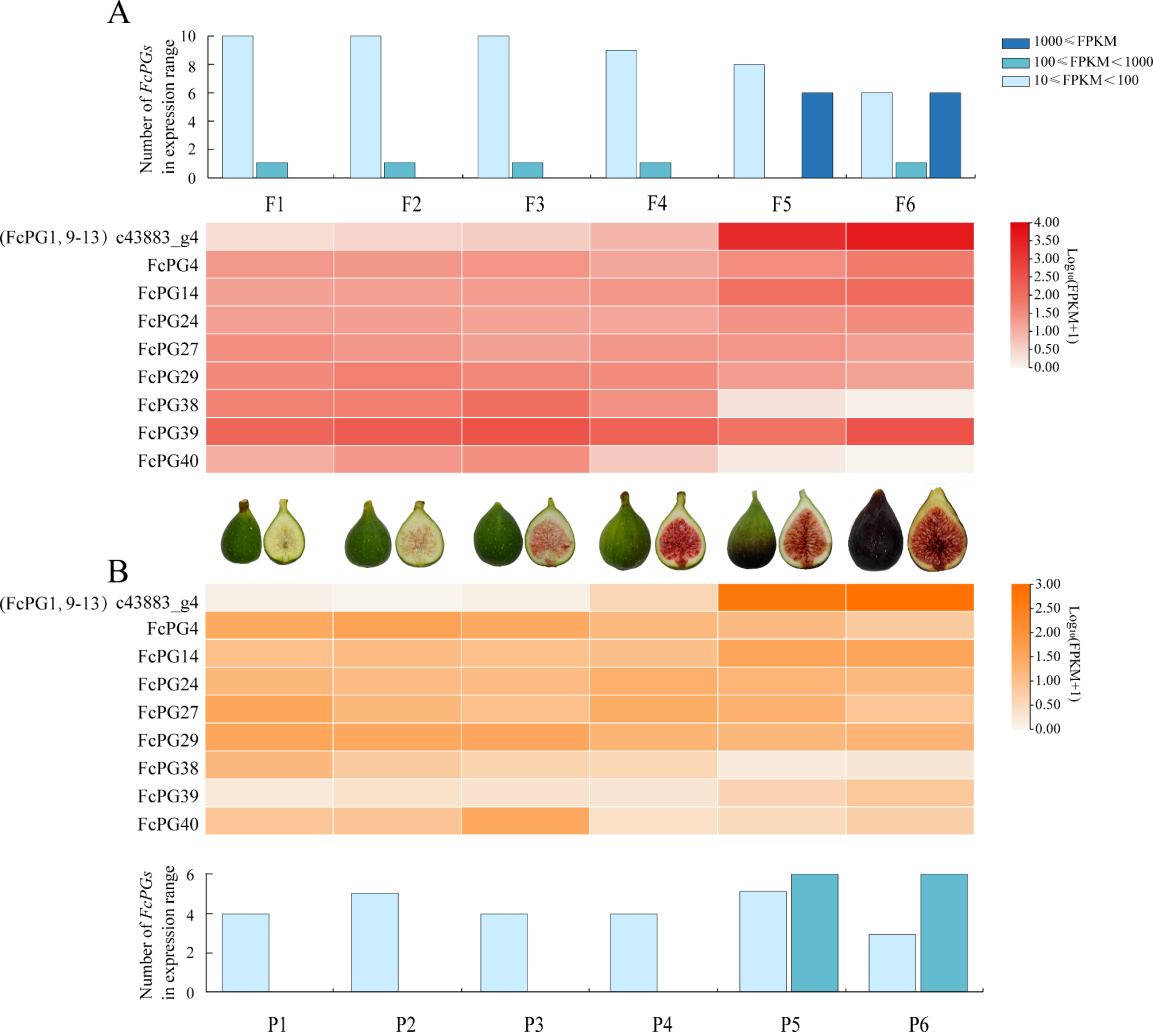
Expression patterns of *FcPG*s during fig fruit development in (**A**) flesh and (**B**) peel. Color scale represents FPKM value normalized by log_10_ counts. The histogram indicates the number of genes in the FPKM expression range. Photographs show fig fruit syconia and internal structure on the six sampling dates

Among the 43 *FcPG* genes, 14 were detected during fig fruit development and ripening (FPKM > 10 in at least one sample) (Fig. 4), and had different expression patterns. *FcPG1*, *9*, *10*, *11*, *12*, *13* and *14* showed upregulation during fruit softening, whereas *FcPG27*, *29* and *38* were downregulated (Fig. 4). The expression of other *FcPG*s fluctuated during fruit development and ripening. The expression of *FcPG39* was rather flesh-specific: it increased in the early stages of fruit development, then decreased, and increased again at fruit ripening, whereas almost no expression was found in the peel (Fig. 4).

**Fig. 5:**
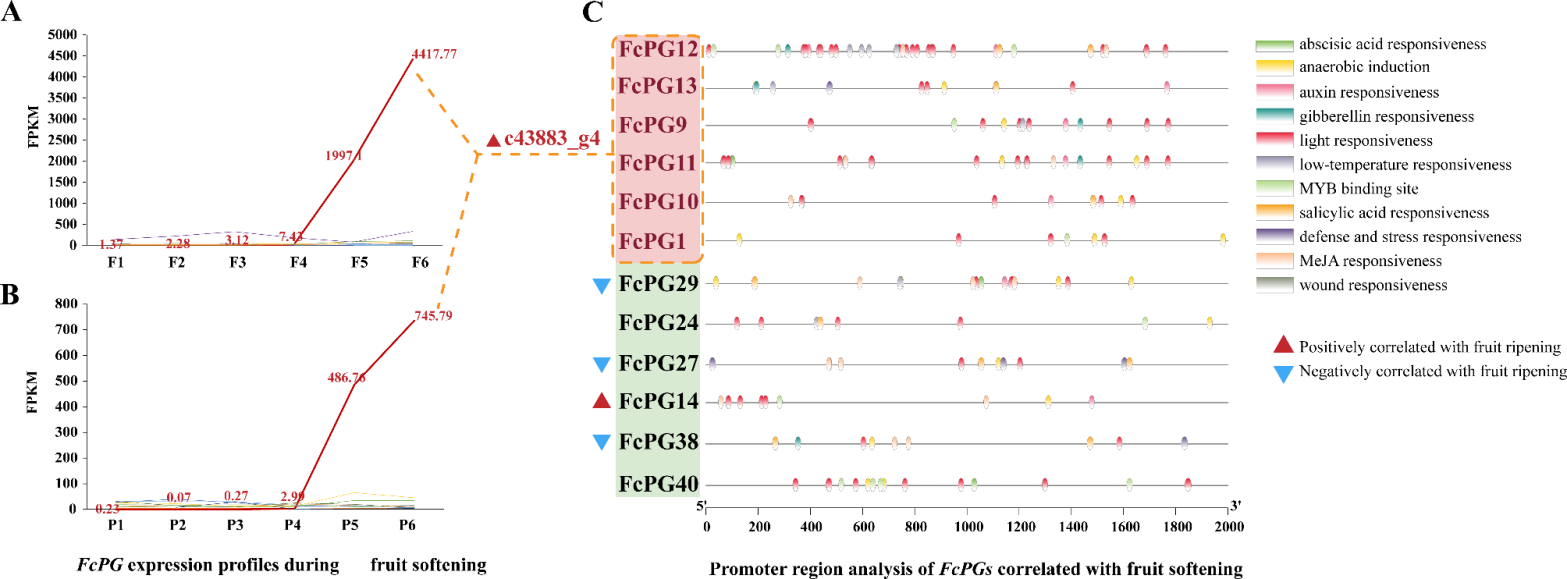
Expression pattern of c43883_g4 and promoter region analysis of *FcPG*s significantly correlated with fruit ripening. Absolute FPKM value of c43883_g4 in (**A**) flesh and (**B**) peel. (**C**) Promoter region analysis of *FcPG*s significantly correlated with fruit ripening

The expression pattern of transcript c43883_g4 (sequence ID in the transcriptome database) was very similar in the flesh and peel of the fig fruit. Almost no expression was detected in the early stage of fruit development, but the expression level increased sharply when the fruit started ripening. The expression of c43883_g4 in the flesh of stage 5 fruit was 268 times higher than that of stage 4 (Fig. 5A). The expression of c43883_g4 was also upregulated (162-fold) in the peel, and upregulation in the flesh and peel continued in stage 6, being 2-fold that in stage 5 (Fig. 5B). The results suggested that the transcript could play an important role in fruit softening. However, c43883_g4 was annotated as a transcript of 6 *PG* genes due to similar sequences in the genome; the *cis*-acting elements of the promoters of transcript c43883_g4 were annotated as 6 predicted *FcPG* genes. Phytohormone-related response elements such as abscisic acid (ABA), light, low temperature and wound signal stress response elements were found, suggesting *FcPG* regulation by phytohormones and a variety of environmental factors. Among them, the light response elements were the most abundant (Fig. 5C).

### Expression patterns of *FcPGs* in fig fruit treated with ethephon

**Fig. 6:**
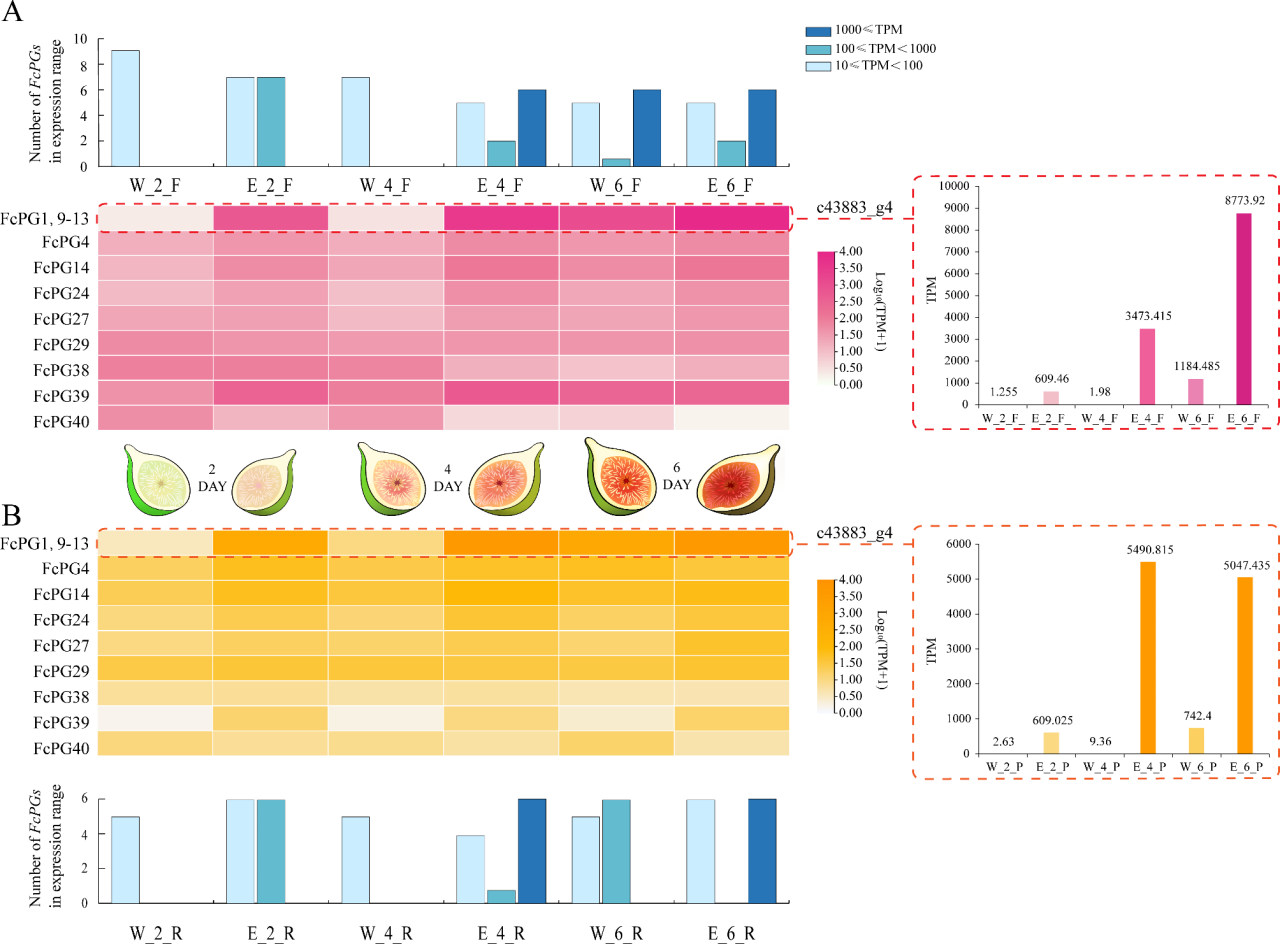
Expression patterns of *FcPG*s in fig fruit upon ethephon treatment in (**A**) flesh, and (**B**) peel. Color scale represents FPKM value normalized by log_10_ counts. Histogram indicates the number of genes in the FPKM expression range. Sketch shows the changes in fig fruit after ethephon treatment

The expression pattern of *FcPG*s in response to ethephon treatment was data-mined (Fig. 6). Ethephon did not induce *FcPG* member expression, and it only affected the 14 *FcPG*s that had been previously detected in the fruit. The expression patterns of *FcPG*s in the flesh and receptacle were consistent in a comparison of treatment time (2, 4 and 6 d) with the control.

After ethephon treatment, 11 *FcPG*s (*FcPG1*, *4*, *9*, *10*, *11*, *12*, *13*, *14*, *24*, *27* and *39*) were upregulated, while *FcPG36* and *40* were downregulated (Fig. 6). The expression of c43883_g4 was upregulated after ethephon treatment, 1700- and 590-fold in the flesh and receptacle, respectively, compared to their corresponding controls 4 d after treatment. The expression difference of c43883_g4 between the treated and control group became smaller 6 d after treatment, as the control fruit entered the ripening stage naturally. The expression of c43883_g4 in the ethephon-treated flesh 6 d after treatment was more than 2-fold that 4 d after treatment (Fig. 6).

### FcPG12 is a major PG in fig fruit softening

**Fig. 7:**
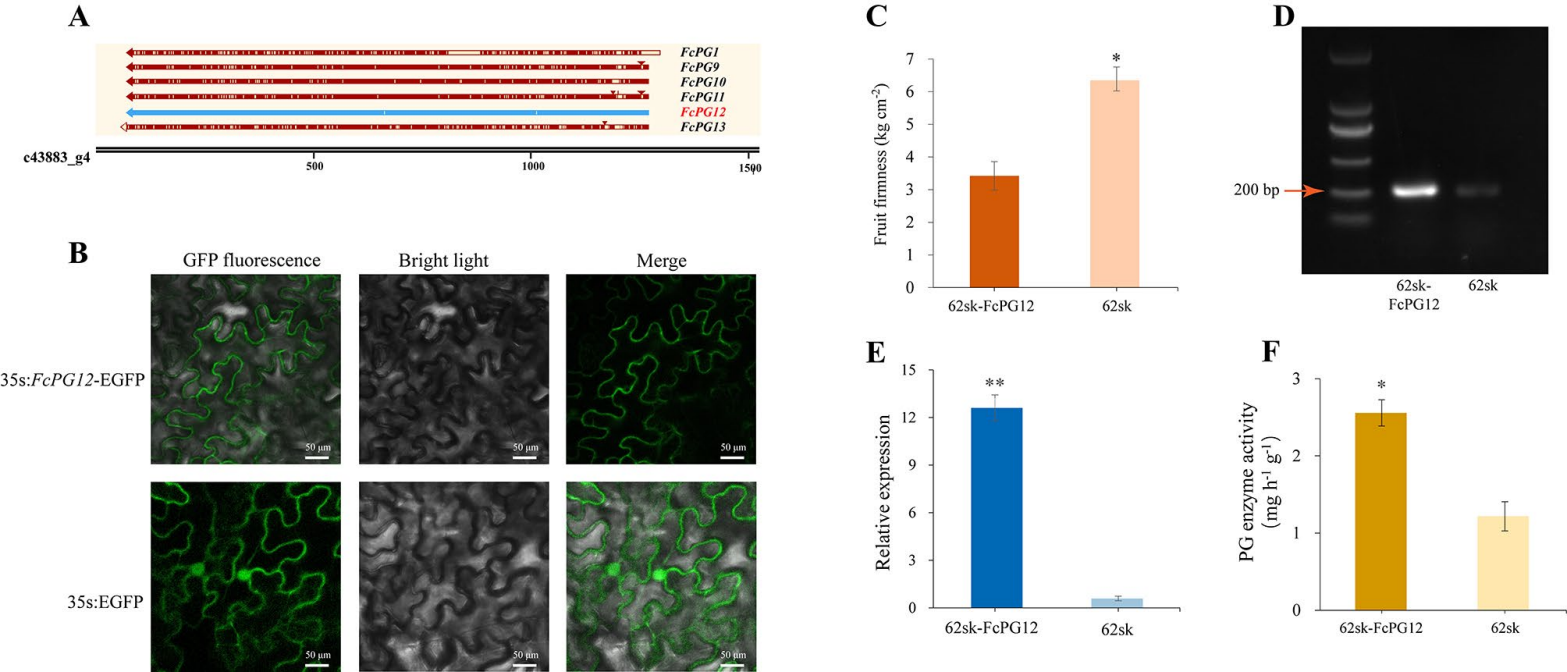
Subcellular localization and transient overexpression of *FcPG12*. (**A**) Sequence alignment of *FcPG1*, *9*, *10*, *11*, *12*, *13* with c43883_g4. (**B**) Subcellular localization of FcPG12–EGFP fusion protein in *N. benthamiana* leaves. Scale bar, 50 μm. (**C**) Fruit tissue firmness with transient overexpression of *FcPG12*. Error bars represent ± SD of three biological replicates. Significance of differences in values (*P < 0.05, **P < 0.01) was determined by Student’s t-test. (**D**) Semi-quantitative RT-PCR and (**E**) qRT-PCR analyses of *FcPG12* expression in fig fruit transiently overexpressing *FcPG12*. The image is an adjacent lane of the same gel (1.8%), and only the image size is cropped. *FcActin* was used as the housekeeping gene. (**F**) PG enzyme activity in fig fruit tissue with transient *FcPG12* overexpression

We cloned *FcPG1*, *9*, *10*, *11*, *12* and *13* and compared their sequences with c43883_g4. A difference of only two single-nucleotide polymorphisms was found between *FcPG12* and c43883_g4 (Fig. 7A). The expression pattern of *FcPG12* during ‘Purple Peel’ fig fruit softening as determined by qRT-PCR was consistent with that of c43883_g4 determined by RNA-Seq (Supplemental Fig. S1). FcPG12 protein was predicted to be located on the cell membrane, and this was verified by fusing *FcPG12* with EGFP under the control of the CaMV 35 S promoter, infiltrating it into leaves of *N. benthamiana* and locating the signal (Fig. 7B).

*FcPG12* and empty plasmid were injected into each of two equal halves of a fig fruit before ripening. Three days later, the firmness of the side with *FcPG12* transient overexpression was lower than that of the control side (Fig. 7C). The relative expression of *FcPG12* at the transcriptional level was higher in the transgenic fruit side than in the control side, as determined by semi-quantitative PCR and qRT-PCR (Fig. 7D, E). PG enzyme activity was also higher in the treated vs. control side (Fig. 7F).

### FcERF5 upregulates *FcPG12* by binding to its promoter

Coexpression pattern analysis revealed 593 and 141 genes with expression patterns similar and opposite to that of *FcPG12*, respectively, by including multiple transcription factors, such as *FcERF*, *FcMYB*, *FcbHLH*. Among them, the expression pattern of *FcERF5* (FCD_00009851) was similar to that of *FcPG12*, increasing sharply when fruits were ripening, as verified by qRT-PCR (Supplemental Fig. S2).

**Fig. 8:**
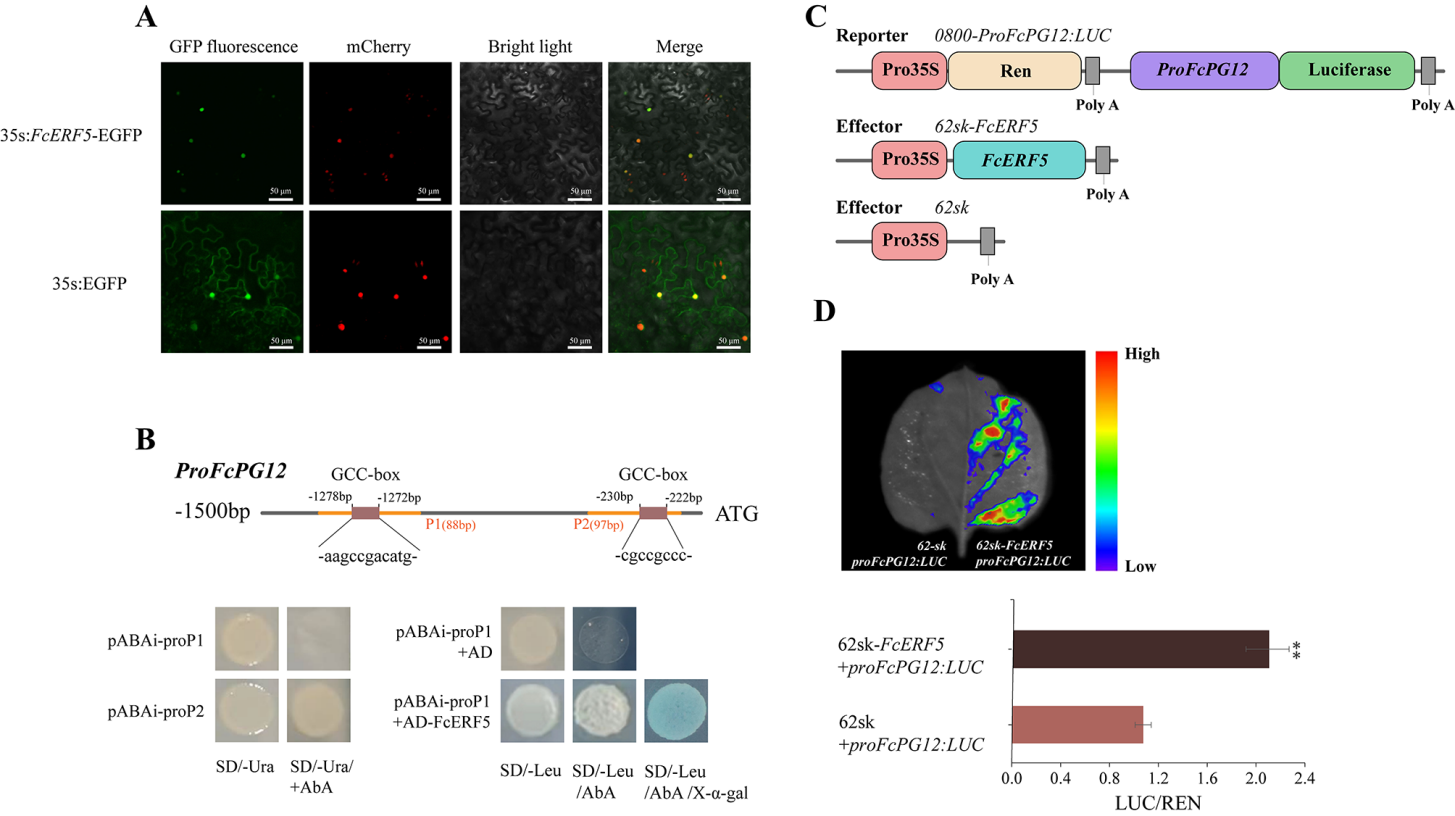
FcERF5 positively regulates *FcPG12* through binding to promoter of FcPG12 (ProFcPG12) in the nucleus. (**A**) Subcellular localization of FcERF5–EGFP fusion protein in mCherry-stained *N. benthamiana* leaves. Scale bar, 50 μm. (**B**) Y1H assay showing FcERF5 binding to the first GCC motif in the *FcPG12* promoter (P1). GCC-box: dehydration-responsive element/C-repeat element. Bold gray line, promoter of FcPG12 (-1500 to -1 bp) used for *cis*-element analysis; bold orange line, *FcPG12* promoter used for Y1H. (**C**, **D**) Dual-luciferase activity assay showing that FcERF5 transactivates the *FcPG12* promoter in *N. benthamiana* leaves. Values are mean ± SD. Imaging of *N. benthamiana* leaves infiltrated with *A. tumefaciens* harboring FcERF5 and *ProFcPG12: LUC*.

The open reading frame (ORF) of *FcERF5* is 906 bp, encoding 301 amino acids with a molecular mass of 32.78 kDa. *FcERF5* contains a conserved AP2/ERF domain and belongs to the fig ERF-Va (IXa) subfamily [29]. Subcellular localization showed FcERF5’s position in the nucleus (Fig. 8A). Two GCC-boxes were predicted on the *FcPG12* promoter (relative profile score threshold > 90%), the first motif ‘aagccgacatg’ located at -1272 bp to -1278 bp upstream of the start codon (ATG), and the second motif ‘cgccgccc’ located -222 to -230 bp upstream of ATG (Fig. 8B). In addition, there was a GCM motif ‘ataacgcaat’ (relative profile score threshold > 95%) that may bind to NAC transcription factors, located at -627 bp to -636 bp upstream of the ATG.

Two GCC-box fragments of the *FcPG12* promoter (P1 and P2) were cloned as respective baits for a Y1H assay. The yeast with *FcPG12* promoter pABAi-P2 grew on the SD/-Ura/+AbA agar plate, indicating that the P2 segment has strong self-activation. Therefore, only P1-positive yeast colonies were selected for the following cotransformation test. The yeast cotransformed with pABAi-P1 and AD-ERF5 plasmids grew on an agar plate containing SD/-Leu/+AbA, and the yeast colony became blue after adding X-α-Gal, whereas the control yeast could not grow on SD/-Leu agar plates, indicating that FcERF5 binds to the first motif fragment of the *FcPG12* promoter (Fig. 8B).

Dual-luciferase reporter assay was performed by transfecting the leaves of *N. benthamiana* with 62sk*-FcERF5* plasmid and *ProFcPG12: LUC* (*FcPG12* promoter) plasmid to measure the LUC activity and detect the luminescence signal (Fig. 8C). The leaf cells of *N. benthamiana* with FcERF5 and *FcPG12* promoter exhibited higher LUC activity than those with 62-sk control and *FcPG12* promoter (Fig. 8D). A strong luminescence signal was detected in the region where FcERF5 and the *FcPG12* promoter were co-injected, but not in the negative controls (Fig. 8D), demonstrating that FcERF5 activates *FcPG12* expression via binding to its promoter. In addition, we determined that FcERF22 (FCD_00010137), from the same cluster as FcERF5, does not bind to the *FcPG12* promoter (Supplemental Fig. S3), suggesting that FcERF5 binding to the *FcPG12* promoter is specific.

### Transient overexpression *FcERF5* in fig fruit

**Fig. 9:**
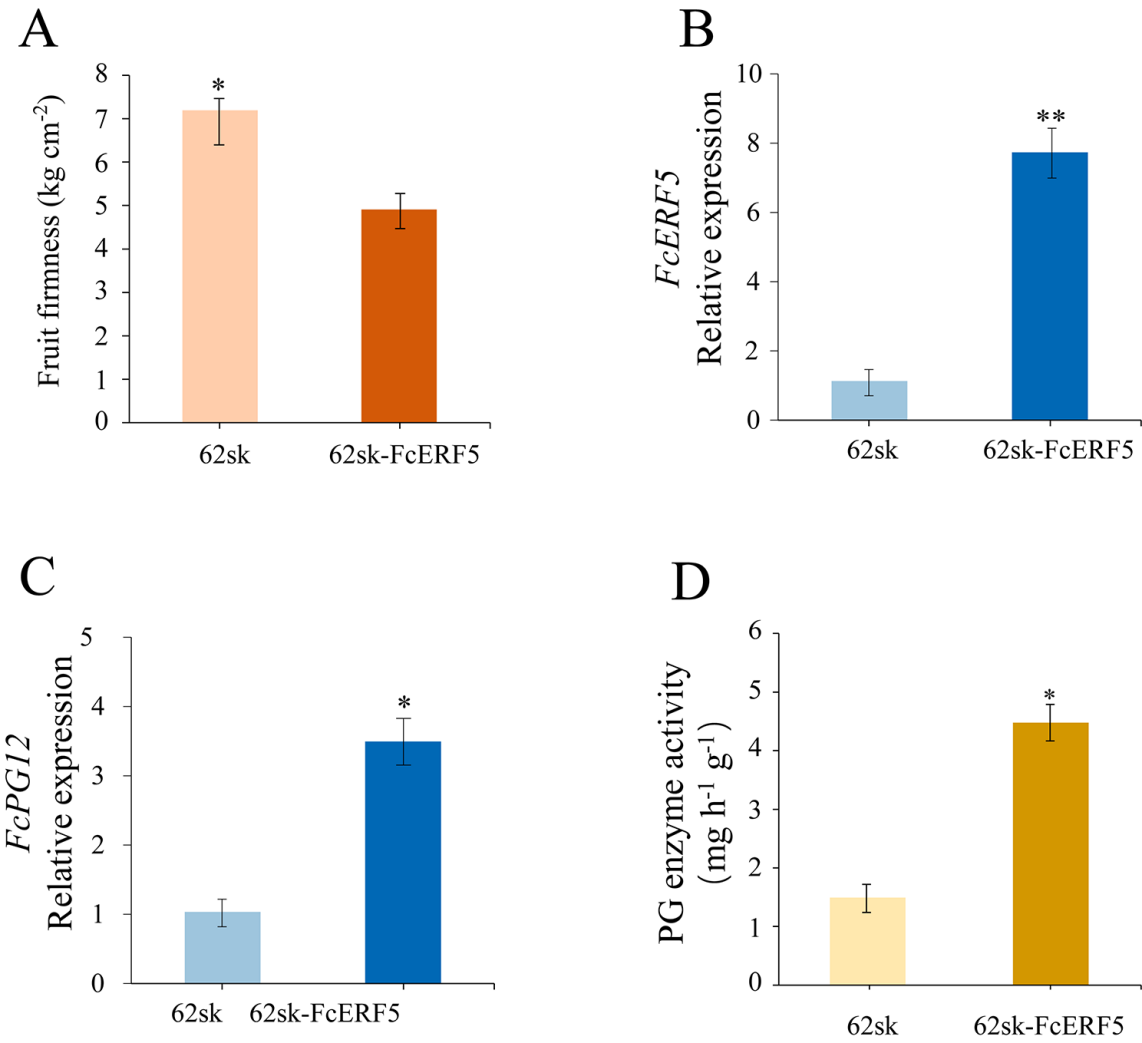
Effect of transient overexpression of *FcERF5* in fig fruit. (**A**) Fruit tissue firmness. Quantitative RT-PCR analysis of (**B**) *FcERF5* and (**C**) *FcPG12* expression. (**D**) PG enzyme activity

The constructed 62sk-FcERF5 plasmid was transiently overexpressed in one half of a fig fruit, and the other half served as a control. The firmness of the fruit half with transient overexpression of *FcERF5* was lower than that of the fruit half with empty vector (Fig. 9A). Based on qRT-PCR analysis, *FcERF5* was upregulated in the fruit tissues of the half with *FcERF5* overexpression (Fig. 9B), verifying the effectiveness of the overexpression at the transcriptional level. The activity of PG enzyme in the receptacle with transient overexpression of *FcERF5* was higher than that of the control (Fig. 9C). The expression of *FcPG12* was upregulated the fruit half with transient overexpression of *FcERF5* (Fig. 9D). Taken together, the results indicate that *FcERF5* activates the expression of *FcPG12*, thereby promoting fig fruit softening.

## Discussion

### FcPG gene family

A total of 43 *PG* genes were identified in *F. carica*, less than the 62 and 54 *PG* genes found in the genomes of *F. microcarpa* and *F. hispida* (Supplemental Table S2), respectively. There were two *FcPG* tandem repeat regions (*FcPG9*, *10*, *11*, *12*, *13* and *FcPG17*, *18*, *19*, *20*, *21*, *22*, *23* gene clusters) on the fig chromosome (Fig. 1A), located on clades C and D of the phylogenetic tree, respectively (Fig. 1B). This is similar to the 28 tomato *PG* genes present in clusters on the chromosome, including five tandem repeat gene clusters [14]. In addition, there were four and three *PG* gene clusters of tandem repeats in *F. microcarpa* and *F. hispida*, respectively (Supplemental Figs. S4 and S5). A comparative collinearity map with *F. pumila* and *F. hispida* was constructed according to the *FcPG9*, *10*, *11*, *12*, *13* gene cluster. Three members in *F. pumila* and four members in *F. hispida* were homologous to the *FcPG*s of this gene cluster, with more dispersed distribution, indicating that tandem duplication occurred on this chromosome segment in fig (Supplemental Fig. S6).

The sequences of *FcPG9*, *10*, *11*, *12*, *13* and *FcPG1* were clustered with the *PG* genes related to fruit softening in apricot (*PaPG*) [30], peach (*PpPG*) [12], *Prunus domestica* (*PdPG1*) [31], pear (*PcPG3*) [32] and papaya [16], suggesting that these *FcPG* genes are related to fruit softening (Fig. 1B). Grape *VvPG1* [33], kiwifruit *CkPG* [34], citrus *CitPG* [35] and apple *MdPG1* [36, 37], with identified roles in fruit softening, were clustered on clade B. *FcPG4*, *31* and *37* were also clustered on clade B. However, only *FcPG4* expression was detected in fig fruit (Fig. 1B). *FcPG4* had opposite expression patterns in fig flesh and peel during fruit development, suggesting its tissue-specific expression (Fig. 4).

Most of the *FcPG*s were not found to be expressed in fig fruit. Approximately one-third of the *FcPG*s were transcribed during fig fruit development and ripening, similar to peach fruit, where only 16 of the 45 *PG* family members were detected [12]. Most of the *FcPG*s which were not expressed in fig fruit, were also not expressed in the profile of the vegetative tissues, i.e., root, leaf and shoot, and they could therefore be pseudogenes, like some of the *Arabidopsis PG*s [6]. The transcript abundance of *FcPG*s which were expressed in fig fruit showed differential patterns during fruit development, and as can be inferred from analysis of the *FcPG* promoter, some *PG* expression may be induced by phytohormones such as ABA or ethylene, together with various stress conditions.

### FcPG12 is a major player in fig fruit texture change during ripening

Among the *FcPG*s expressed in the fruit, the transcript c43883_g4 demonstrated unique expression characteristics. There was almost no expression of c43883_g4 in young fruit, but it increased rapidly and continuously from the start of ripening, when its level became much higher than that of the other *FcPG*s (Fig. 4). Moreover, c43883_g4 was induced by ethephon (Fig. 6). Transcript c43883_g4 was annotated as the gene cluster *FcPG9*, *10*, *11*, *12*, *13* and phylogenetically clustered as *FcPG1*. In general, only one gene in the tandem repeat region shows relatively high expression, whereas the other genes have either low expression or no transcript product at all [6]. In our study, *FcPG12* was most similar to the transcript c43883_g4 (Fig. 7A). In addition, only *FcPG12* transcripts were identified when five specific primers were respectively used to detect the transcripts of the five *PG* genes (*FcPG9*–*13*). Pure *FcPG12* without the SNP (single-nucleotide polymorphism) base of the amplified transcripts was validated by multiple clone sequencing, and the presence of only single-peak melting curves in qRT-PCR. Taken together, that *FcPG12* is the major member transcribed during fruit development in this gene cluster.

Blast alignment analysis indicated that FcPG12 and FcPG39 in this study correspond to the sequences of FcPG1 and FcPG2, respectively, that were previously reported and published in GenBank [28]. In the phylogenetic tree, *FcPG12* is clustered on clade C, with strong homology to the key *PG*s of other species on this clade. However, it has a distant relationship with clade B (*FcPG39* is in this clade), which also carries many key fruit softening-related *PG*s (Fig. 1B). It therefore seems that the key *PG*s involved in fruit softening in different species are on different phylogenetic clades. FcPG12, with a relative molecular mass of 4.18 kDa and pI of 8.73 is similar to the key PG proteins of other species, such as PpPG, PaPG and PdPG1, collected in this paper (Fig. 1B). Sequence similarity analysis showed that the PG domains were highly conserved, but the PG protein sequences varied greatly among the different species (Supplemental Fig. S7). To obtain evidence that *FcPG12* affects fruit softening, we performed transient overexpression of *FcPG12* in fig. Increased PG enzyme activity, together with decreased firmness of the receptacle revealed that *FcPG12* plays an important role in fruit softening by degrading pectin (Fig. 7). This is similar to the results of *AaPG18* overexpression in kiwifruit [15].

Identification of key *PG* genes is the basis for improving fruit storage and extending shelf life. CRISPR/Cas9 technology was used to obtain 4-bp and 10-bp deletions and a 1-bp insertion in the *SlPG* gene in tomato. Frameshift mutation of *SlPG* led to early termination of amino acid translation, thus delaying fruit softening [38]. In another study, virus-induced gene silencing technology was used to interfere with the expression of the peach β-galactosidase gene, resulting in decreased expression of *PpPG21* and *PpPME3* and their corresponding enzyme activities, which inhibited the softening of peach fruit [39]. Interference of *MdPG1* in ‘Royal Gala’ apple resulted in decreased pectin-degradation rate and increased fruit firmness [40]. As the key glycoside hydrolase in figs, modulating the expression of *FcPG12* may be an important way to slow down the rapid fruit softening and improve its texture.

### The expression regulation of FcPG12 by transcription factors

Understanding *FcPG12* expression regulation is of great significance for analyzing the mechanism of softening during fig ripening. The expression pattern of *FcERF5* during fruit softening was similar to that of *FcPG12*, increasing when the fig fruit started to ripen (Supplemental Fig. S2). FcERF5 is closely clustered with AtERF5. According to similarity prediction in the SWISS-PROT protein database, FcERF5 is a transcription factor that can bind to the GCC-box and may be a transcription activator involved in signal-transduction pathways regulating gene expression. Through Y1H and dual luciferase reporter assays, we verified that FcERF5 functions as an activator of *FcPG12* by binding to the *FcPG12* promoter (Fig. 8). This is in agreement with a recent report that PpERF/ABR1 promotes *PpPG* expression in peach by binding to the *PpPG* promoter [41]. Subcellular localization of FcERF5 supported occurrence of the regulation process in the nucleus (Fig. 8A). Fruit firmness and PG enzyme activity after overexpression of *FcERF5* were similar to that following *FcPG12* overexpression, providing further evidence that FcERF5 upregulates *FcPG12* and promotes fruit softening (Fig. 9).

AP2/ERF is a large family that includes many members related to fruit ripening and softening in Fig. [29]. *FcERF62* also had expression characteristics similar to those of *FcPG12*. *FcERF62* (FCD_00028202) belongs to FcERF IV subfamily. The expression of *FcERF62* gradually increased with fruit development. At fruit ripening, it reached its highest abundance in the fruit flesh (FPKM of 100), while its expression level was lower in the peel (highest FPKM was 12). The expression of *FcERF62* increased in fruit treated with ethephon. However, direct regulation of *FcPG12* by *FcERF62* requires further verification.

In addition to these *FcERF*s, weighted gene coexpression network analysis (WGCNA) showed that *FcMYB108*, *FcMYB4*, *FcbHLH137*, *FcbHLH149* and *FcNAC29* in coexpression module ‘MEcyan’ and ‘MEblack’ had expression characteristics similar to those of *FcPG12* (Supplemental Fig. S8). *RhMYB108*, which is highly homologous to *FcMYB108*, has been reported to be involved in ethylene- and jasmonic acid-induced petal senescence in roses [42]. NAC gene family members play an important role in ethylene and ABA signal-transduction pathways. They are involved in the fine regulation of cell wall modification genes and play a role in regulating texture changes during fruit ripening and softening [1]. AtNAP, which is closely related to FcNAC29, may function in the transition between active cell division and cell expansion [43]. MYB and NAC transcription factor binding sites were predicted on the *FcPG12* promoter. However, more experimental evidence is required to reveal the panorama of *FcPG12*-expression regulation.

## Conclusion

In this study, 43 *FcPG*s were identified in the fig genome for the first time. According to their gene structure, motif and *cis*-acting element characteristics, they were assigned to seven groups (A–G). Based on RNA-Seq data analysis, gene cloning and sequence comparison, *FcPG12* was isolated as a key *PG* related to fruit softening. The function of *FcPG12* in promoting fruit softening was confirmed by transient overexpression and qRT-PCR analyses. FcERF5 binds to the *FcPG12* promoter in the nucleus and promotes its expression, resulting in fruit softening, as proven by subcellular localization, Y1H, dual luciferase and transient expression experiments. Our results provide a basis for exploring the molecular mechanism of fig softening, and an understanding of how ERF regulates cell wall-modifying *PG* gene transcription during fruit softening.

## Materials and methods

### Plant materials

Five-year-old common figs (*Ficus carica* var. 108B) were used for gene isolation and transient genetic transformation. The figs were grown at Shangzhuang Experimental Station of China Agricultural University, Beijing, with 3 × 3 m spacing and a vertical trellis system. The development of fig fruit was divided into six stages. Fruits of similar maturity, uniform size and clean surface were selected. For each sample collection, the flesh, peel and receptacle of fruits were separated to freeze in liquid nitrogen and store at -80 °C separately for further use.

### Identification of *FcPG* gene family members

*F. carica* genome data were downloaded from NCBI (DDBJ/EMBL/GenBank access code: VYVB01000000) [44]. Fig *PG* genes were obtained by homologous blast of *Arabidopsis* PG protein sequences and HMM search of the PG conserved domain in the Pfam database (http://pfam.xfam.org/). *Arabidopsis* PG protein sequences were downloaded from TAIR (https://www.arabidopsis.org). Fig PGs were searched using BlastP (e-value threshold ≤ 1e-5) with Tbtools and AtPG protein sequences as queries [45]. The Hidden Markov Model (HMM) file of the PG protein domain (PF00295) was download from the Pfam database. Sequences containing PG protein domains were retrieved from the fig genome database using HMMER 3.0 [46]. Combining the results of the two screening methods, protein sequences without PG conserved motifs and short proteins (< 50 amino acids) were eliminated. The retained protein sequences were verified to contain PG domains by NCBI Batch-CD. The relative molecular weights and isoelectric points (pIs) of the predicted PGs were calculated by ExPASy (https://www.expasy.org/). Prediction of signal peptides was carried out by SignalP (https://services.healthtech.dtu.dk/service.php?SignalP-5.0).

### Chromosomal location, gene duplication, phylogenetic tree construction and PG sequence analysis

Gene structure visualization of the GFF3 file of the fig genome was conducted using TBtools [45]. All PG protein sequences of fig and *Arabidopsis thaliana* were aligned with Clustal W [47], and a phylogenetic tree was constructed by MEGA 6.0 software using the maximum likelihood (ML) method with 1000 replicates [48]. Multiple sequence alignment was done with Clustal X software and ESPript online website (https://espript.ibcp.fr/ESPript/cgi-bin/ESPript.cgi). Conserved motifs were analyzed using MEME (https://meme-suite.org/meme/tools/meme). The *FcPG* promoter (2000-bp region upstream of the start codon of each gene) was extracted from the fig genome and submitted to the PlantCare database (http://bioinformatics.psb.ugent.be/webtools/plantcare/html/) to determine putative *cis-*elements. Transcription factor binding sites were predicted by JASPAR (https://jaspar.genereg.net/). Corresponding coding regions were aligned using Clustal W, and the number of non-synonymous substitutions per non-synonymous site (Ka) and the number of synonymous substitutions per synonymous site (Ks) were calculated by KaKs Calculator 2.0 [49]. The time of gene duplication was determined by the formula T = Ks/2r, where r is the divergence rate of nuclear genes. In *A. thaliana*, r was considered to be a synonymous substitution of 7 × 10^− 9^ per site per year [50]. Collinear analysis of *Ficus carica*, *Ficus hispida*, and *Ficus pumila* was performed using the multicollinear scanning toolkit (MCScanX) [51].

### *FcPG* expression during fruit ripening

The expression pattern of *FcPG*s was analyzed by re-mining two RNA-Seq libraries of fig fruit established in our laboratory. The first library consisted of data on ‘Purple Peel’ fig fruit development (SRA login: PRJNA723733) [52]. The RNA-Seq data were generated and the gene-expression counts were normalized to fragments per kilobase of transcript per million mapped reads (FPKM). The second library contained data on the flesh and receptacle of figs after ethephon treatment [53]. Briefly, ‘Brown Turkey’ fig fruit before ripening were injected with 1 mL of 250 mg L^− 1^ ethephon, and the flesh and receptacle of the fruit were collected 2, 4, and 6 d after treatment for transcriptome sequencing. The gene-expression counts were normalized to transcripts per million (TPM).

### Quantitative reverse transcription (qRT)-PCR validation of gene expression

Total RNA was extracted from the receptacle of fig fruit by cetyltrimethylammonium bromide (CTAB) [54], and RNA was reverse-transcribed to cDNA using a reverse transcription kit (TaKaRa Biotechnology, Dalian, China). The primer pairs were designed by Primer3 (https://bioinfo.ut.ee/primer3/). The qRT-PCR was performed on an Applied Biosystems QuantStudio 6 Flex system, using TB Green® Premix Ex Taq (RR420Q, TaKaRa). Each sample was run in three technical repeats. The reaction conditions were as follows: 40 cycles of 95 °C for 30 s, 95 °C for 5 min, and 60 °C for 34 s. The *FcActin* gene was used as an internal control [55], and gene relative expression was determined by the 2^−ΔΔCT^ method [56]. All primers are listed in Supplemental Table S1.

### Subcellular localization

Subcellular localization was predicted using the online tool Cell-PLoc 2.0 (http://www.csbio.sjtu.edu.cn/bioinf/Cell-PLoc-2/). The gene primers were designed by SnapGene 4.2.4 software. The full-length gene’s coding sequence (CDS) was cloned from cDNA and inserted into the binary vector pBI121 with enhanced green fluorescent label (EGFP). The 35 S:FcPG12-EGFP, 35 S:FcERF5-EGFP and control vectors were introduced into *Agrobacterium tumefaciens* strain GV3101 by heat shock. *Agrobacterium* cells were cultured on Luria broth (LB) agar plates containing rifampicin (25 µg mL^− 1^) and kanamycin (50 µg mL^− 1^) at 28 °C for 2–3 d. For each transformation experiment, a single colony was cultured in 5 mL LB containing antibiotics at 28 °C for 16 h. The culture was centrifuged at 6000 g and the pellet was resuspended in 25 mL medium (10 mM MES, pH 5.7, 100 µM Acetosyringone (AS), 10 mM MgCl_2_) to OD_600_ of 0.6–0.8. After standing at 28 °C for 2 h, the suspension was infiltrated into 4-week-old *Nicotiana benthamiana* leaves [57]. Fluorescent signal was observed 48–72 h later using a confocal laser-scanning microscope (Olympus FV3000, Tokyo, Japan).

### Transient genetic transformation in fig fruit

The full-length CDSs of *FcPG12* and *FcERF5* were cloned into vector pGreenII 62-sk [58] and introduced into *Agrobacterium* strain GV3101 for transient overexpression in fig fruit. Using a transient genetic transformation method for Fig. [59], the fruits before ripening (stage 4 in Song et al., 2021) were cut into two equal parts and one half was injected with *Agrobacterium* with the recombinant plasmids. The other half served as a control. After 3 d, fruit hardness was measured and the fruit receptacle tissue was collected. Around 60 fruits were injected for each gene.

### Fruit firmness and PG enzyme measurements

Fruit firmness was measured with AICE digital fruit hardness tester GY-4, and each measurement was repeated three times. PG enzyme activity was measured using PG enzyme activity kit G0701F (Grace Biotechnology, Suzhou, China).

### Yeast one hybrid (Y1H) assay

The full-length CDS of *FcERF5* was inserted into vector pGBKT7-AD (AD-TF vector, Clontech, Mountain View, CA, USA), and the *FcPG12* promoter fragment with the GCC-box domain was inserted into vector pABAi. The constructed pABAi-ProFcPG12 plasmid was transformed into the Y1H Gold yeast strain by PEG/LiAC method and grown on an agar plate with SD growth medium without uracil (SD/-Ura). After 3 d, the positive yeast colony was transferred to an SD/-Ura agar plate and screened to determine the minimum inhibitory concentration of aureobasidin A (AbA) (50–500 µg L^− 1^) for yeast growth. Then the constructed pAD-FcERF5 and pABAi-ProFcPG12 plasmids were co-transformed into yeast strains, and these were inoculated on SD plates without leucine (SD/-Leu) containing X-α-Gal to observe color changes.

### Dual-luciferase reporter assay

The *FcPG12* promoter fragment (1500 bp region upstream of the start codon) was cloned and inserted into the pGreenII 0800-LUC vector [58], and introduced into *Agrobacterium* strain GV3101. The *Agrobacterium* with constructed 62sk-FcERF5 and 0800-ProFcPG12 plasmids (1:9) was co-transformed into *N. benthamiana* leaves by infiltration. After 48 h, 1 mM fluorescein was sprayed on the leaves, and luciferase imaging was performed using LUCK2019 (Vilber Lourmat, Paris, France). Luciferase activities (LUC and REN) were measured using a GloMax® -20/20 signal tube luminescence meter (Promega, Madison, WI, USA), and the ratio of the two was used as the calculated result.

### Weighted gene coexpression network analysis (WGCNA)

WGCNA of the RNA-Seq data (Sect. 2.4) was performed using R package ‘WGCNA’ [60]. The RNA-Seq data covering the top 75% of the variance were screened, and the recommended threshold was automatically calculated. Genes with expression pattern similarity higher than 90% were selected.

### Statistical analysis

Statistical analyses, consisting of one-way ANOVA followed by Duncan’s new multiple range test and Student’s *t*-test, were performed with IBM SPSS Statistics Version 20.0.

## Electronic supplementary material

Below is the link to the electronic supplementary material.

Supplementary Material 1

Supplementary Material 2

## Data Availability

All data and supporting materials can be found within the manuscript. Accession numbers of sequence data from this article can be found in the GenBank data libraries: FcPG12 (OQ982383), FcERF5 (OQ982384), CkPG (L12019), CmPG1 (AF062465), CmPG2 (AF062466), CitPG (EF185420), CpPG1 (FJ007644), CpPG4 (GQ479796), MaPG3 (AY603339), MdPG1 (L27743), PaPG (ADT82706), PcPG1 (AB084461), PcPG3 (BAF42034), PdPG1 (DQ375247), PpPG (EF568784), PpPG1 (BAH56488), PpPG2 (CAA54448), TAPG1 (NM001246866), TAPG2 (U70480), TAPG4 (U70481), VvPG1 (AY043233).
