# Supplementary material for: Polygalacturonase gene family analysis identifies FcPG12 as a key player in fig (Ficus carica L.) fruit softening
Source: BMC Plant Biol. 2023 Jun 14;23:320. doi: 10.1186/s12870-023-04315-7 (PMC10265768; doi:10.1186/s12870-023-04315-7)
Supplement: Supplementary file 2 — Supplementary Material 2 [file 12870_2023_4315_MOESM2_ESM.pdf]

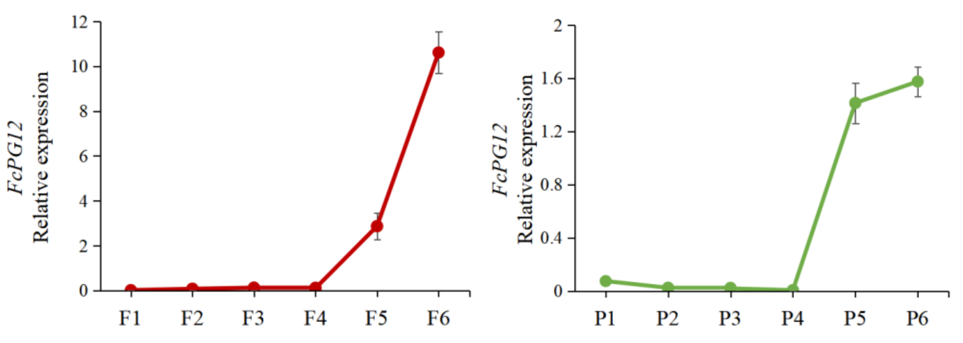

Supplemental Figure S1. Expression pattern of *FcPG12* in the flesh (left) and peel (right) during fig fruit softening.

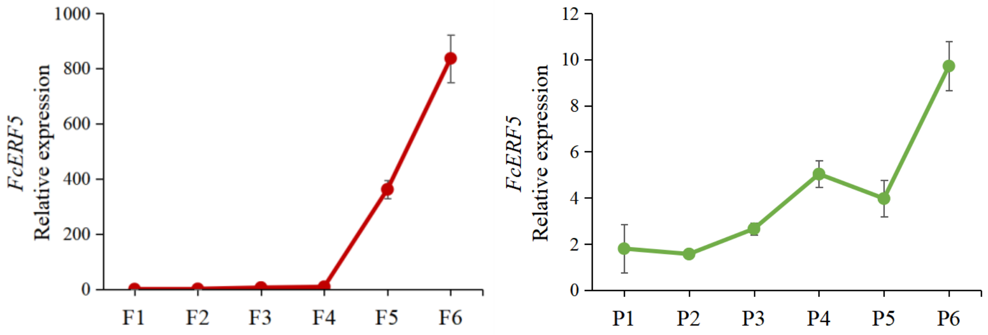

Supplemental Figure S2. Expression pattern of *FcERF5* in the flesh (left) and peel (right) during fig fruit softening.

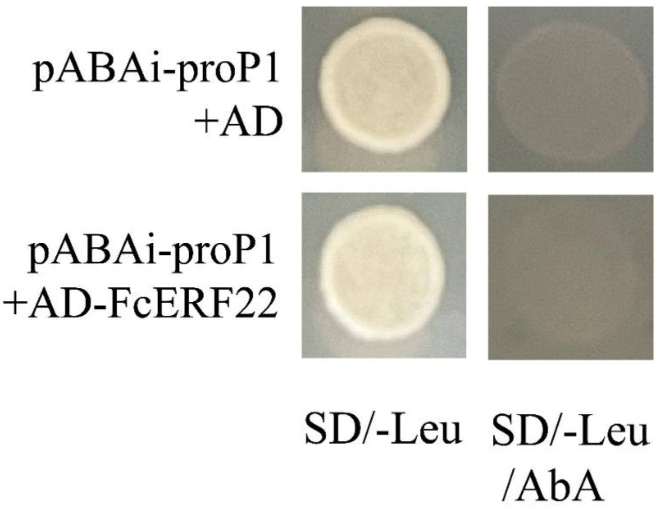

Supplemental Figure S3. Y1H assay showing that FcERF22 does not bind to the first GCC motif in the *FcPG12* promoter (P1).

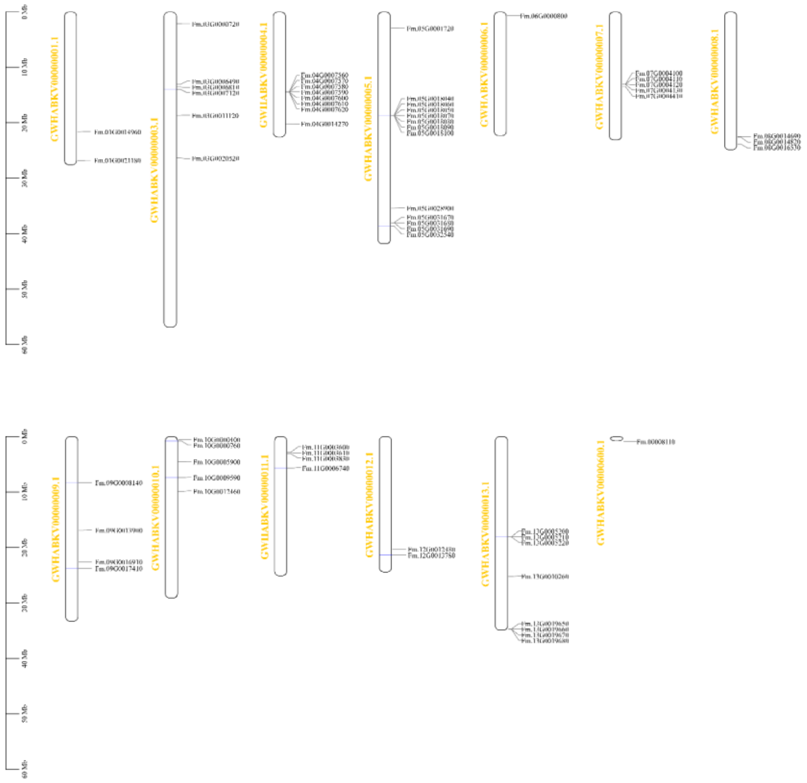

Supplemental Figure S4. Chromosomal distribution of *PG* genes of *F. microcarpa*.

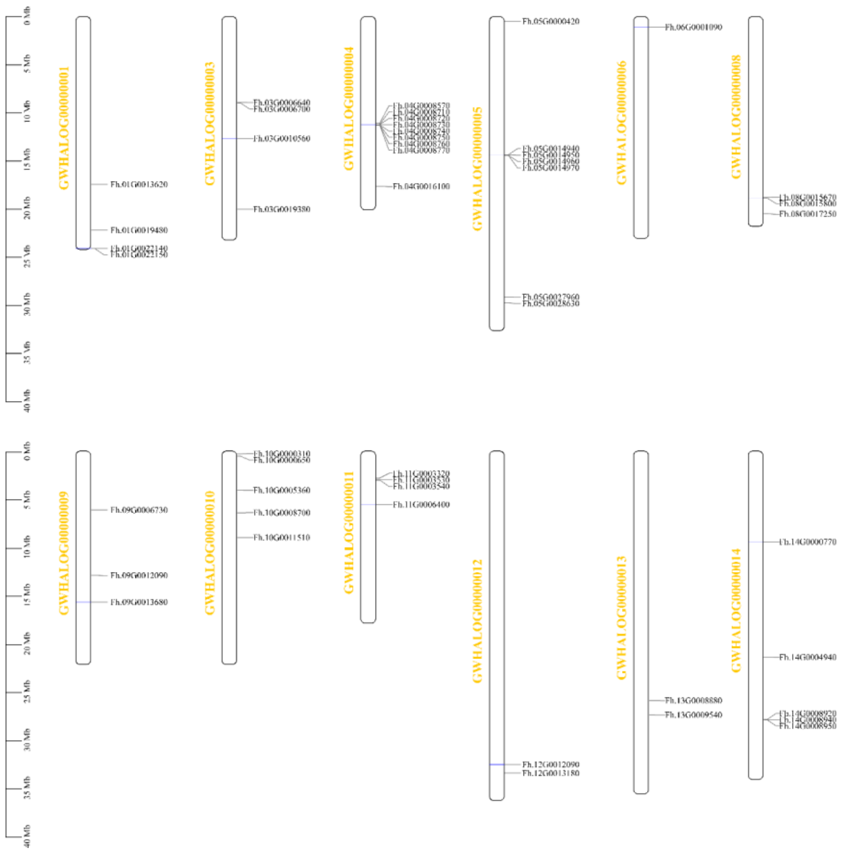

Supplemental Figure S5. Chromosomal distribution of *PG* genes of *F. hispida*.

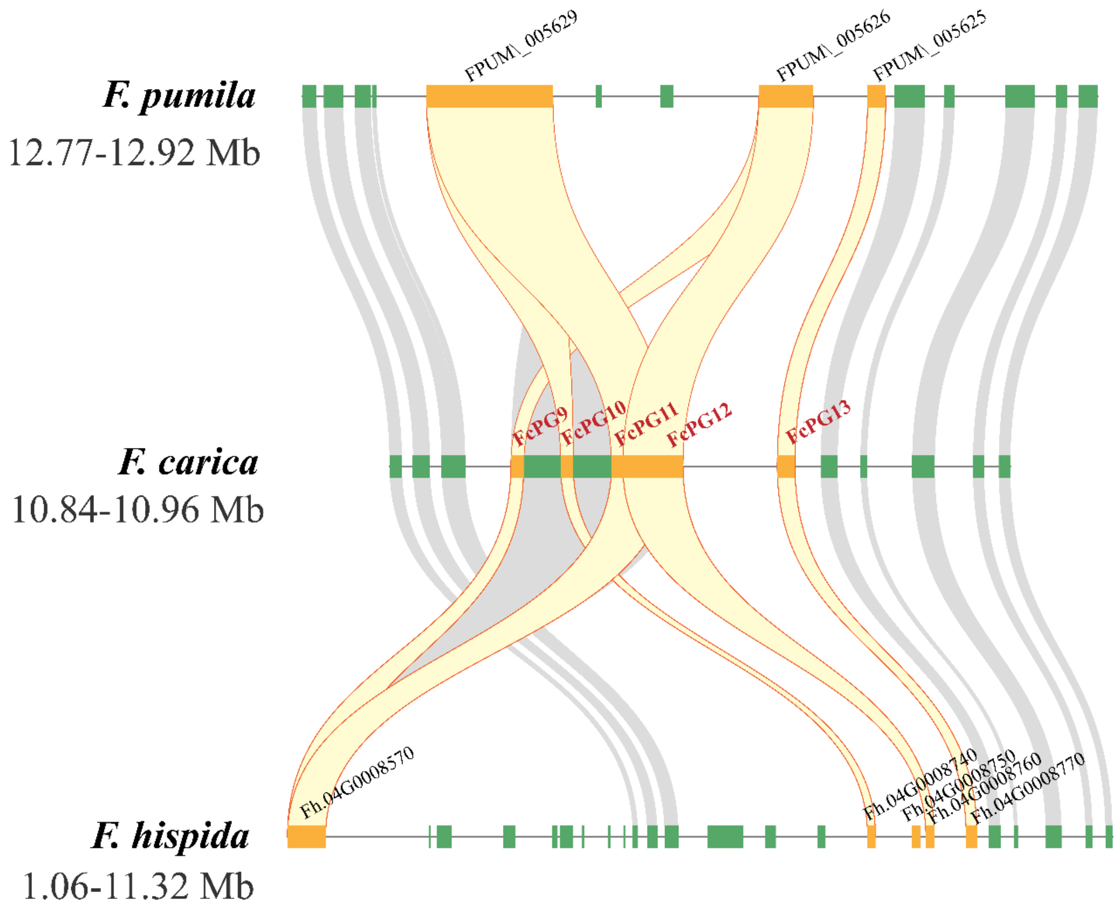

Supplemental Figure S6. Collinearity relationship of the *FcPG9, 10, 11, 12, 13* gene cluster among *Ficus pumila, F. carica*, and *F. hispida*. Identified collinear genes are linked by yellow blocks.

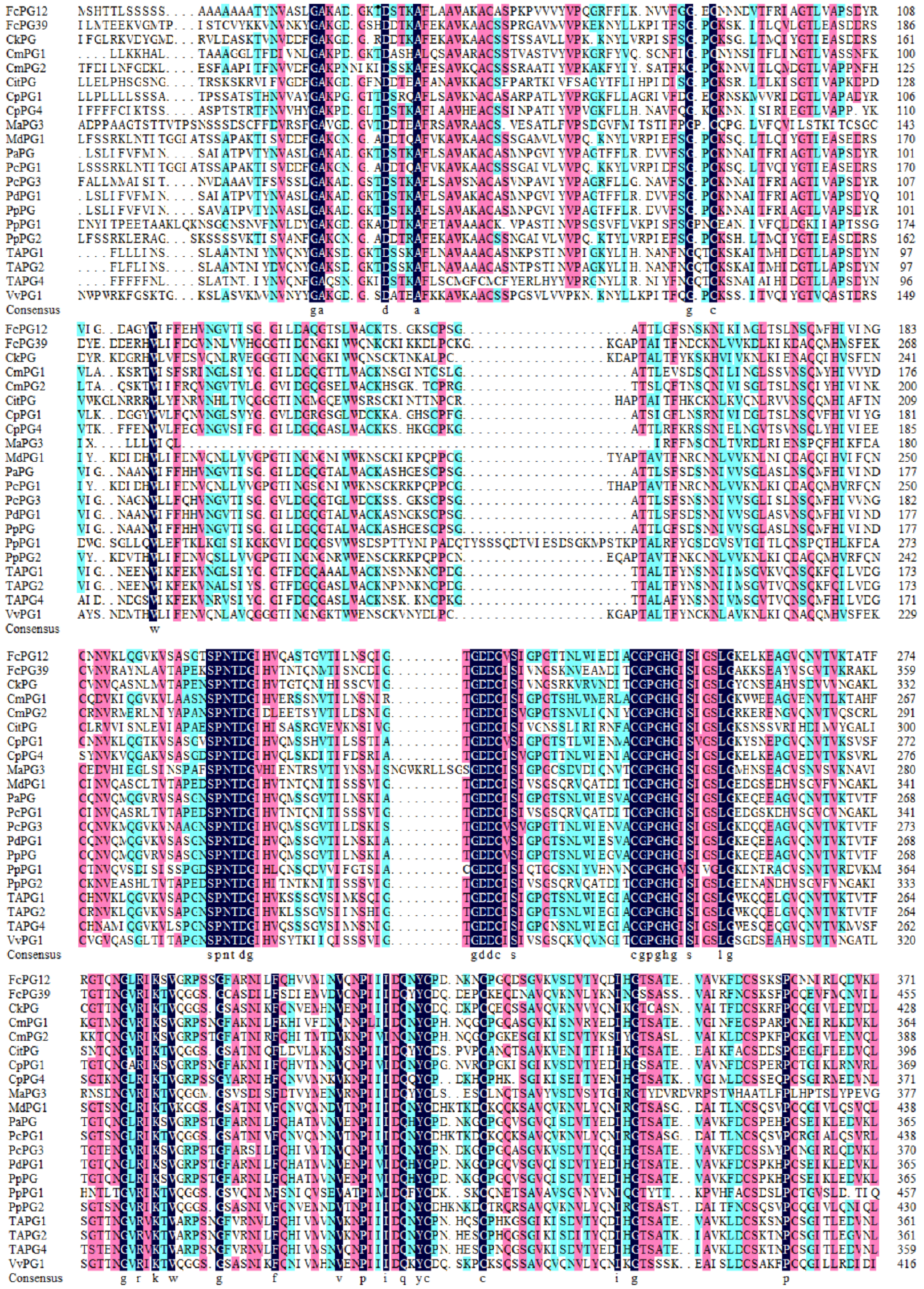

Supplemental Figure S7. Multiple sequence alignment of FcPG12 and FcPG39 with PGs of other species.

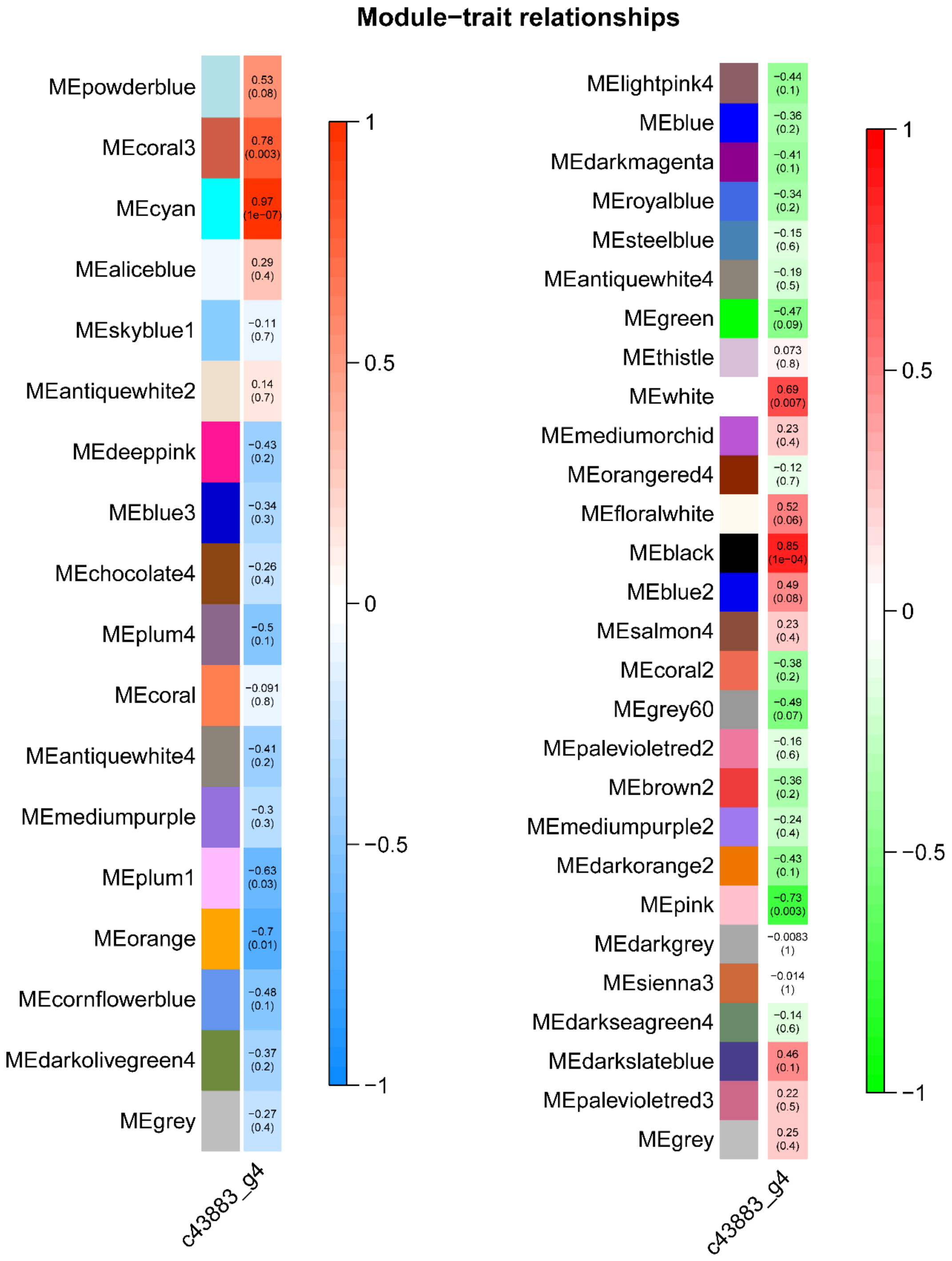

Supplemental Figure S8. WGCNA indicates that coexpression modules ‘MEcyan’ and ‘MEblack’ are significantly positively correlated with c43883_g4. (A) Correlation heatmap of expression patterns of c43883_g4 and other transcripts during fruit ripening and softening. (B) Correlation heatmap of expression patterns of c43883_g4 and other transcripts after ethephon treatment.

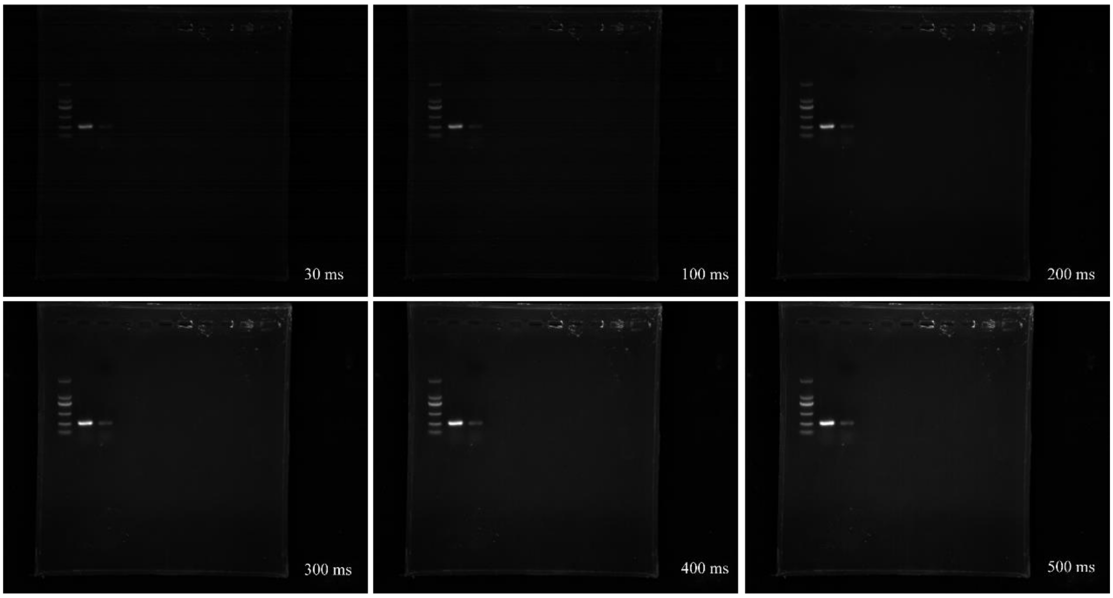

Supplemental Figure S9. The full length original and uncropped gel of overexpressing *FcPG12* semi-quantitative RT-PCR analyses in multiple exposures. The exposure time was 30 ms to 500 ms.
